# Signal mining of botulinum toxin type A adverse events based on FAERS database and its implications for the treatment of cerebral palsy

**DOI:** 10.3389/fnhum.2025.1676051

**Published:** 2025-11-18

**Authors:** Huajie Wang, Shiyu Ma, Jun Lai, Yubin Huang

**Affiliations:** 1 Department of Pharmacy, Ganzhou People's Hospital, Ganzhou, Jiangxi, China; 2 The Second Affiliated Hospital of Guangzhou University of Chinese Medicine, Guangdong Provincial Hospital of Chinese Medicine, Guangzhou, Guangdong, China; 3 State Key Laboratory of Dampness Syndrome of Chinese Medicine, Guangzhou, Guangdong, China; 4 Department of Rehabilitation Medicine, Ganzhou People's Hospital, Ganzhou, Jiangxi, China

**Keywords:** FDA adverse event reporting system, neuromuscular blocker, botulinum toxin type A, cerebral palsy, adverse drug reactions, signal mining

## Abstract

**Objective:**

Based on the US Food and Drug Administration Adverse Event Reporting System (FAERS), signal mining of adverse drug events (AEs) caused by Botulinum Toxin Type A (BoNTA) was performed to explore its safety implications for the treatment of cerebral palsy (CP).

**Methods:**

The OpenVigil 2.1 platform was used to extract AE reports on BoNTA from the FAERS database, covering the period from the fourth quarter of 2003 to the second quarter of 2024. Safety data were analyzed using the Reporting Odds Ratio (ROR) and Proportional Reporting Ratio (PRR), with BoNTA designated as the primary suspect drug.

**Results:**

A total of 124,538 AE reports related to BoNTA were identified, showing an overall upward trend in the annual report counts. Most reports originated from the United States, with patients predominantly aged 36–60 years and predominantly female. Prolonged hospitalization was the most frequently reported serious adverse event. Signal analysis identified 325 disproportionately reported events across 21 system-organ classes (SOCs). The top five preferred terms (PTs) by frequency were eyelid ptosis, dysphagia, muscle weakness, blurred vision, and injection site swelling. The top five PTs based on signal strength were brow ptosis, Mephisto sign, botulism, bizarre personal appearance, and neuromuscular toxicity. Notable lowest-level terms (LLTs) included eye swelling, injection site edema, facial pain, facial discomfort, increased residual urine volume, blurred vision, and eyelid swelling.

**Conclusion:**

In clinical practice involving BoNTA for CP treatment, clinicians should pay close attention to these identified signals. Strengthened pre-injection evaluation and post-injection monitoring are recommended to enable early detection and timely intervention, ensuring medication safety for patients.

## Introduction

Cerebral palsy (CP) refers to a group of disorders caused by non-progressive injuries to the developing brain of the fetus or infant, leading to persistent impairments in movement and posture (Paul et al., 2022; te Velde et al., 2019). Epidemiological studies show that 2–3 out of every1,000 live births are affected by CP (Vitrikas et al., 2020), with spasticity being the most common form, accounting for 60–82% of cases (Khandaker et al., 2019; Jonsson et al., 2019). It is primarily characterized by muscle spasms, increased muscle tone, and stiffness (Walhain et al., 2021). Currently, there is no cure for CP and rehabilitation therapy remains the primary treatment approach. However, the treatment process is lengthy, and the therapeutic effects are often slow or limited.

Neuromuscular blocking agents (NMBAs), also known as muscle relaxants, are capable of inhibiting the normal binding of the neurotransmitter acetylcholine to its receptors, thereby blocking nerve-muscle conduction and alleviating spasticity (Selinger et al., 2022; Lee et al., 2021). Botulinum toxin type A (BoNTA) is a NMBA produced by *Clostridium botulinum*. In December 1989, the U. S. FDA approved BoNTA as a new drug for nationwide use. Koman et al. (1993) first employed BoNTA to treat children with CP. In recent years, BoNTA has gained widespread use in clinical practice for local injection treatment of spastic CP, offering the advantages of rapid onset of action and convenience. A single injection of BoNTA provides long-lasting effects, positively contributing to the recovery of limb motor function and creating favorable conditions for rehabilitation by relaxing muscles (Klein et al., 2024). However, due to the need for repeated injections, the clinical safety of BoNTA has raised significant concerns, with an increase in reports of adverse events (AEs). However, these reports were mostly individual cases and lacked a comprehensive analysis.

The U. S. FDA Adverse Event Reporting System (FAERS) is an open-source database that collects information on drug reactions reported by patients. It plays an important role as a primary data source for signal mining studies on AEs and provides essential insights for safety monitoring and risk assessment (Li et al., 2023; Shi et al., 2024). Therefore, based on the FAERS database, this study systematically mined BoNTA-related AE signals, aiming to analyze their risk characteristics and provide key data support and clinical implications for the safe application of BoNTA in the treatment of cerebral palsy.

## Materials and methods

### Research materials

This study used OpenVigil 2.1 platform^1^ to extract AE reports from the FAERS database. The search terms encompassed the generic name, brand name, and ATC code of the drug, along with keywords such as “Botulinum toxin type A,” “AbobotulinumtoxinA,” “Botulinum A neurotoxin,” “Botulinum antitoxin type A,” “AGN 191622,” and “ANT-1207.” Data extraction covered the period from the 4th quarter of 2003 to the 2nd quarter of 2024. AE reports were excluded if they were duplicate, non-drug-related, or contained uncertain drug names.

### Research objectives

The objectives of this study were to collect and analyze statistical data on reporting time, reporting country, patient age, and sex in AE reports and to examine safety data in which the target drug was identified as the primary suspected cause. Medical Dictionary for Regulatory Activities (MedDRA, version 26.1) was used to standardize the lowest-level terms (LLTs) of AEs into preferred terms (PTs). AE reports corresponding to the same PT were counted and combined and classified by system organ class (SOC) for further analysis.

### Signal detection

Signal detection was performed using the reporting odds ratio (ROR) and proportional reporting ratio (PRR). The frequency of target AE associated with the target drug was compared with the background frequency. When the number of AE reports (a) ≥ 3, a risk signal is considered present if the lower limit of the 95% confidence interval (CI) for the two-tailed ROR test is > 1, or if the PRR ≥ 2 and *χ*^2^ ≥ 4. The detailed calculation methods for the ROR are outlined in Tables 1, 2.

**Table 1:** Disproportionate fourfold contingency table.

Project	Targeted AE reports	Other AE reports	Total
Targeted Drug	a	b	a + b
Other Drugs	c	d	c + d
Total	a + c	b + d	n = a + b + c + d

a, number of reports containing the target drug of target AEs; b, number of reports containing other AEs of the target drug; c, number of reports containing the target AEs of other drugs; d, number of reports containing other drugs of other AEs.

**Table 2:** Calculation formulas and thresholds for ROR and PRR.

Methods	Calculation formula	Threshold
ROR	ROR= $\frac{\left(a/c\right)}{\left(b/d\right)}$	lower limit of 95% CI > 1, a ≥ 3
	SE(lnROR) = $\sqrt{\left(\frac{1}{a}+\frac{1}{b}+\frac{1}{c}+\frac{1}{d}\right)}$	
	95% CI = $e^{\ln(\mathrm{ROR})}\pm 1.96\sqrt{\frac{1}{a}+\frac{1}{b}+\frac{1}{c}+\frac{1}{d}}$	
PRR	PRR = $\frac{a/(a+b)}{c/(c+d)}$	a ≥ 3, PRR ≥ 2, and χ^2^ ≥ 4
	SE(InPRR) = $\sqrt{\left(\frac{1}{a}+\frac{1}{a+b}+\frac{1}{c}+\frac{1}{c+d}\right)}$	
	95% CI = $e^{\ln(\mathrm{ROR})}\pm 1.96\sqrt{\frac{1}{a}+\frac{1}{a+b}+\frac{1}{c}+\frac{1}{c+d}}$	

a, number of reports containing the target drug of target AEs; b, number of reports containing other AEs of the target drug; c, number of reports containing the target AEs of other drugs; d, number of reports containing other drugs of other AEs; 95% CI, 95% confidence interval; ROR, reporting odds ratio; PRR, proportional reporting ratio.

## Results

### Composition of AE reports

**Figure 1:**
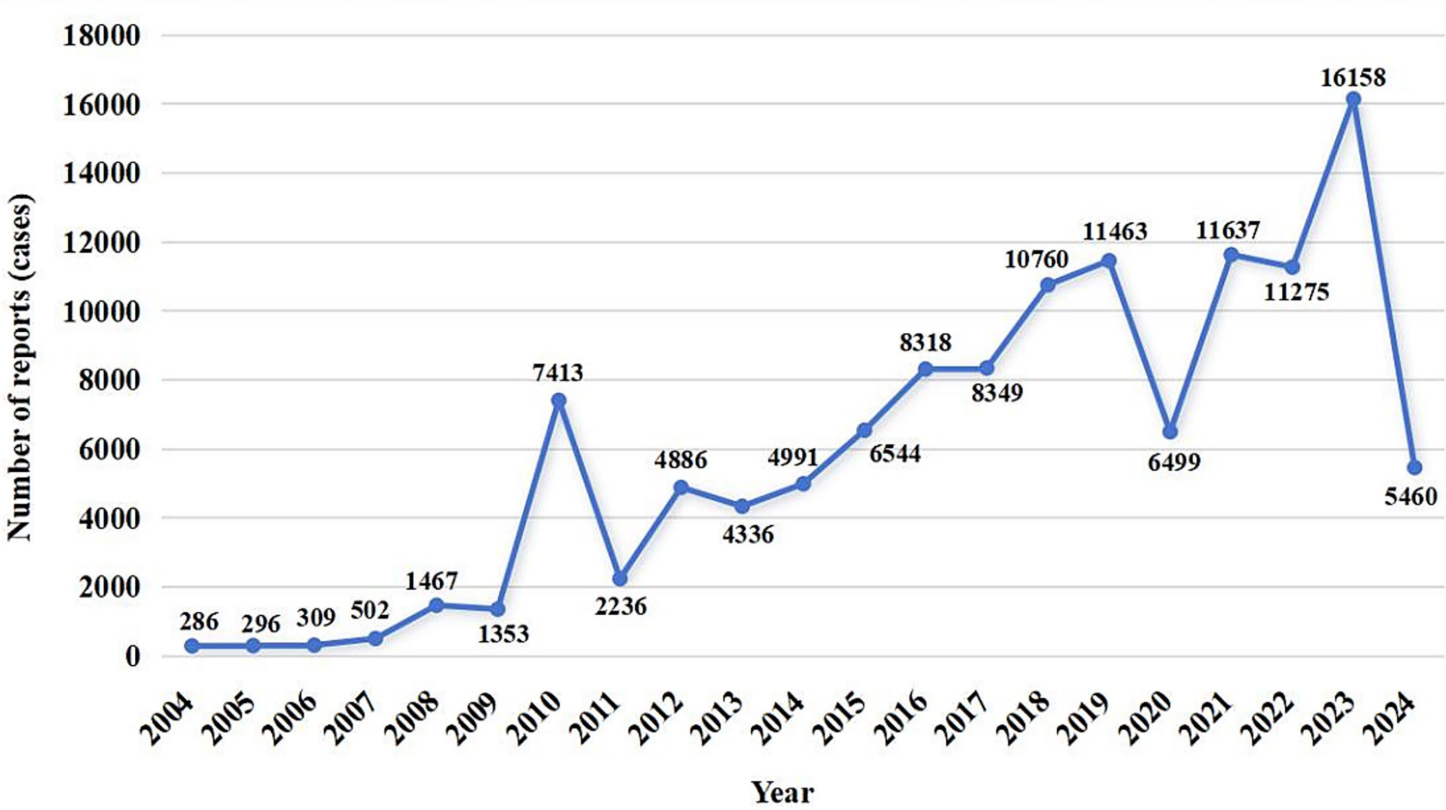
Annual distribution of AE reports related to BoNTA.

**Figure 2:**
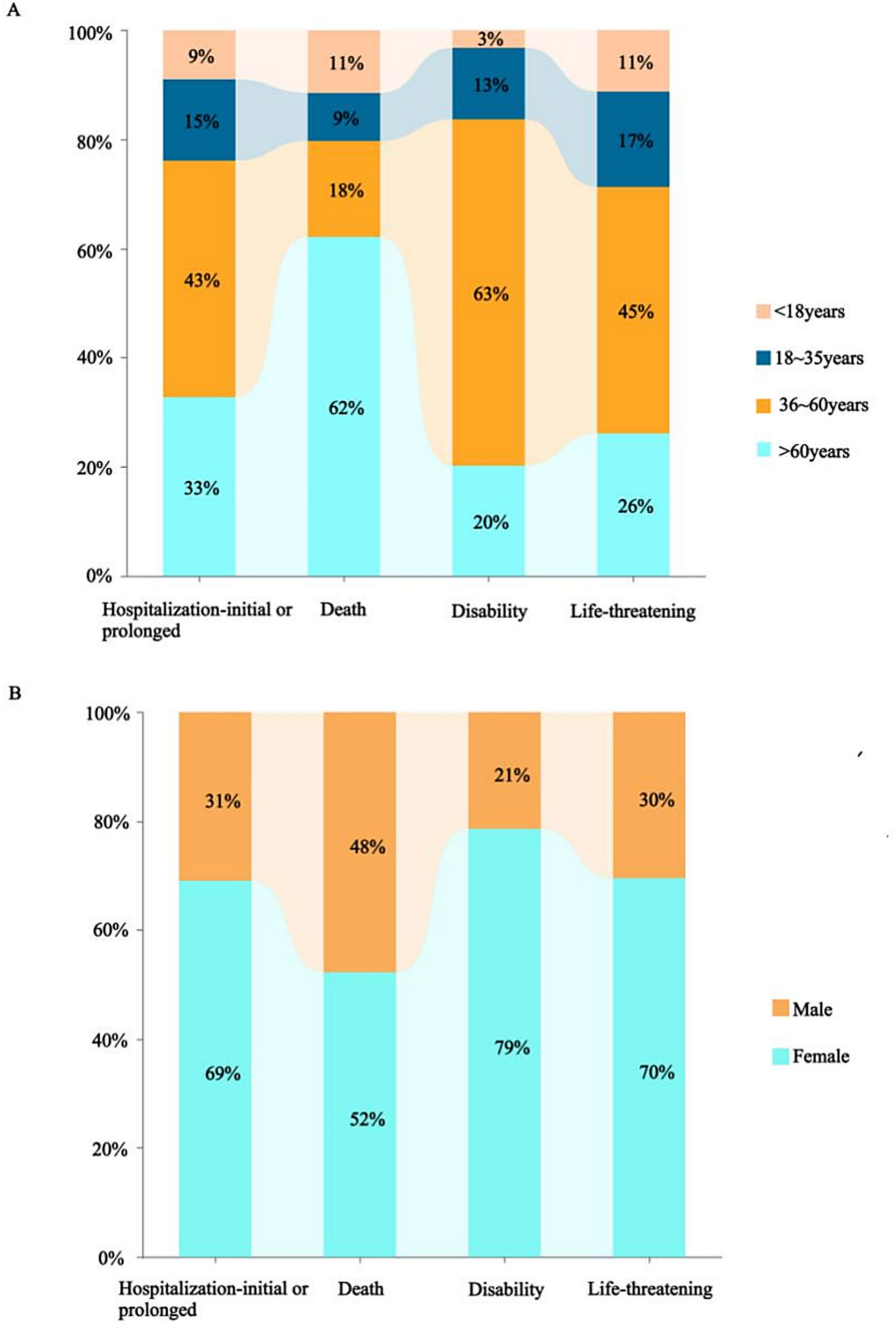
Relationship between severe adverse outcomes and age **(A)** or gender **(B)**.

A total of 12,631,150 AE reports were retrieved, of which 124,538 were associated with BoNTA across therapeutic and aesthetic indications, accounting for approximately 0.99% of the total. As shown in Figure 1, the number of AE reports related to BoNTA showed an overall upward trend from 2004 to 2024; data on BoNTA usage were unavailable and therefore not presented. A more pronounced increase occurred after 2014, peaking by 2023. As shown in Table 3, the majority of AE reports related to BoNTA came from the United States (85.15%), followed by Japan (2.02%), and Canada (1.92%). Excluding missing data, the age group with the highest number of reports was 36–60 years (25.81%). Regarding gender distribution, a higher number of reports were submitted by females (74.88%) than by males (11.44%). The most commonly reported serious adverse outcomes were prolonged hospitalization/extended hospital stay (6.52%), followed by death (2.02%), disability (1.94%), and life-threatening events (0.58%). As shown in Figure 2, the primary groups that experienced prolonged hospitalization, disability, and life-threatening conditions were individuals aged 36–60 years and female. In contrast, the majority of those who died were over 60 years old, with a nearly equal sex distribution between males and females.

**Table 3:** Basic information on AEs reported in cases receiving BoNTA from the FAERS database.

Characteristics	Case numbers (*n*)	Case proportion (%)
Reporting country/region		
United States	106,040	85.15
Japan	2,513	2.02
Canada	2,392	1.92
Other/Missing information	13,593	10.91
Age		
<18 years	2,114	1.70
18–35 years	7,036	5.65
36–60 years	32,147	25.81
>60 years	10,729	8.62
Missing information	72,512	58.22
Gender		
Male	14,249	11.44
Female	93,250	74.88
Missing information	17,039	13.68
Severe adverse outcomes		
Hospitalization - initial or prolonged	8,114	6.52
Death	2,521	2.02
Disability	2,410	1.94
Life-threatening	722	0.58

### Analysis of adverse event reports by system organ class (SOC)

A total of 325 disproportionately reported events were identified in the signal analysis of AE reports in which BoNTA was the primary suspected drug, involving 78,278 cases. BoNTA exerted a certain influence on 21 SOCs, as shown in Table 4. The top five SOCs, according to the number of reports, were general disorders and administration site conditions: injury, poisoning, procedural complications, eye diseases, nervous system disorders, musculoskeletal and connective tissue disorders.

**Table 4:** Distribution of adverse events (AEs) and disproportionately reported events by system organ class (SOC) in the FAERS database.

SOC	AE reports (*n*)	Disproportionately reported events (*n*)
General disorders and administration site conditions	38,231	61
Injury, poisoning and procedural complications	21,455	31
Eye disorders	6,961	58
Nervous system disorders	3,985	55
Musculoskeletal and connective tissue disorders	2,955	19
Gastrointestinal disorders	1,556	14
Skin and subcutaneous tissue disorders	959	14
Respiratory, thoracic and mediastinal disorders	676	19
Infections and infestations	353	8
Renal and urinary disorders	326	11
Product issues	239	10
Pregnancy, puerperium and perinatal conditions	214	2
Investigations	100	7
Ear and labyrinth disorders	93	3
Social circumstances	90	1
Surgical and medical procedures	41	5
Vascular disorders	19	2
Metabolism and nutrition disorders	10	1
Psychiatric disorders	7	2
Blood and lymphatic system disorders	5	1
Reproductive system and breast disorders	3	1

### Analysis of adverse event reports by preferred term (PT)

**Figure 3:**
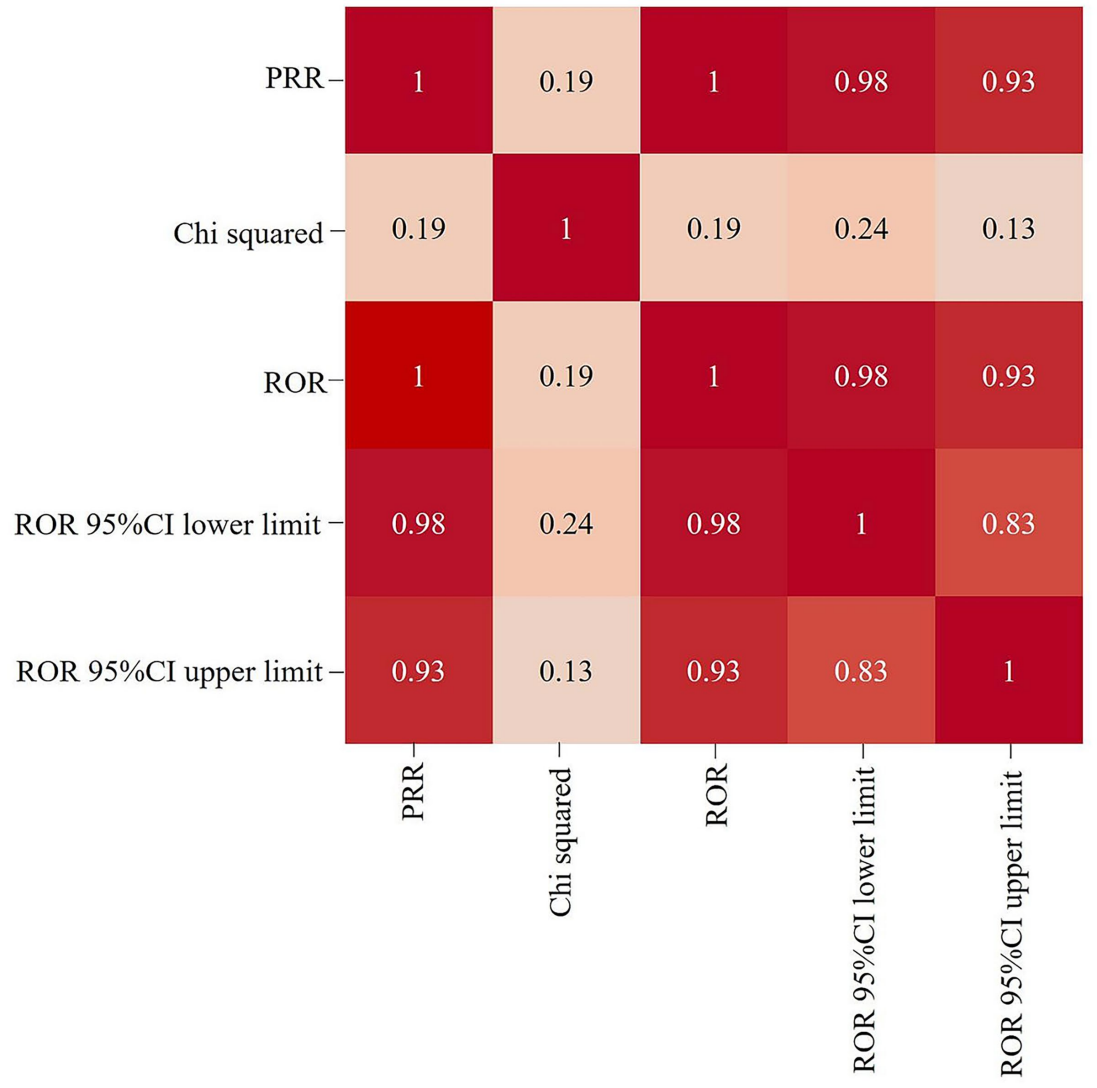
Heat map showing the correlation among signal detection methods for BoNTA adverse events (AEs). ROR, reporting odds ratio; PRR, proportional reporting ratio; 95% CI, 95% confidence interval.

**Figure 4:**
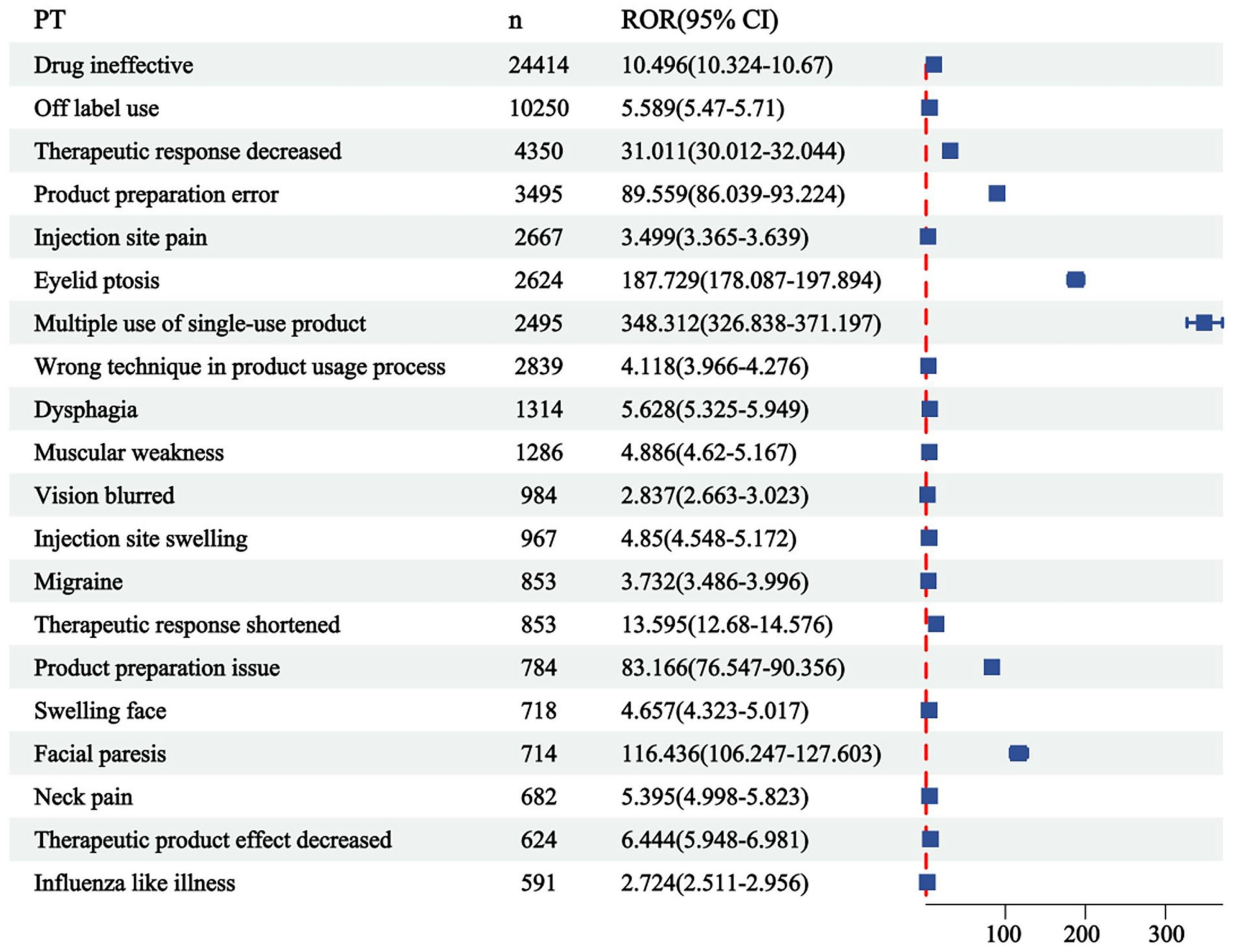
Top 20 most frequent adverse events (AEs) for BoNTA at the preferred terms (PTs) level from FAERS. ROR, reporting odds ratio; 95% CI, 95% confidence interval.

**Figure 5:**
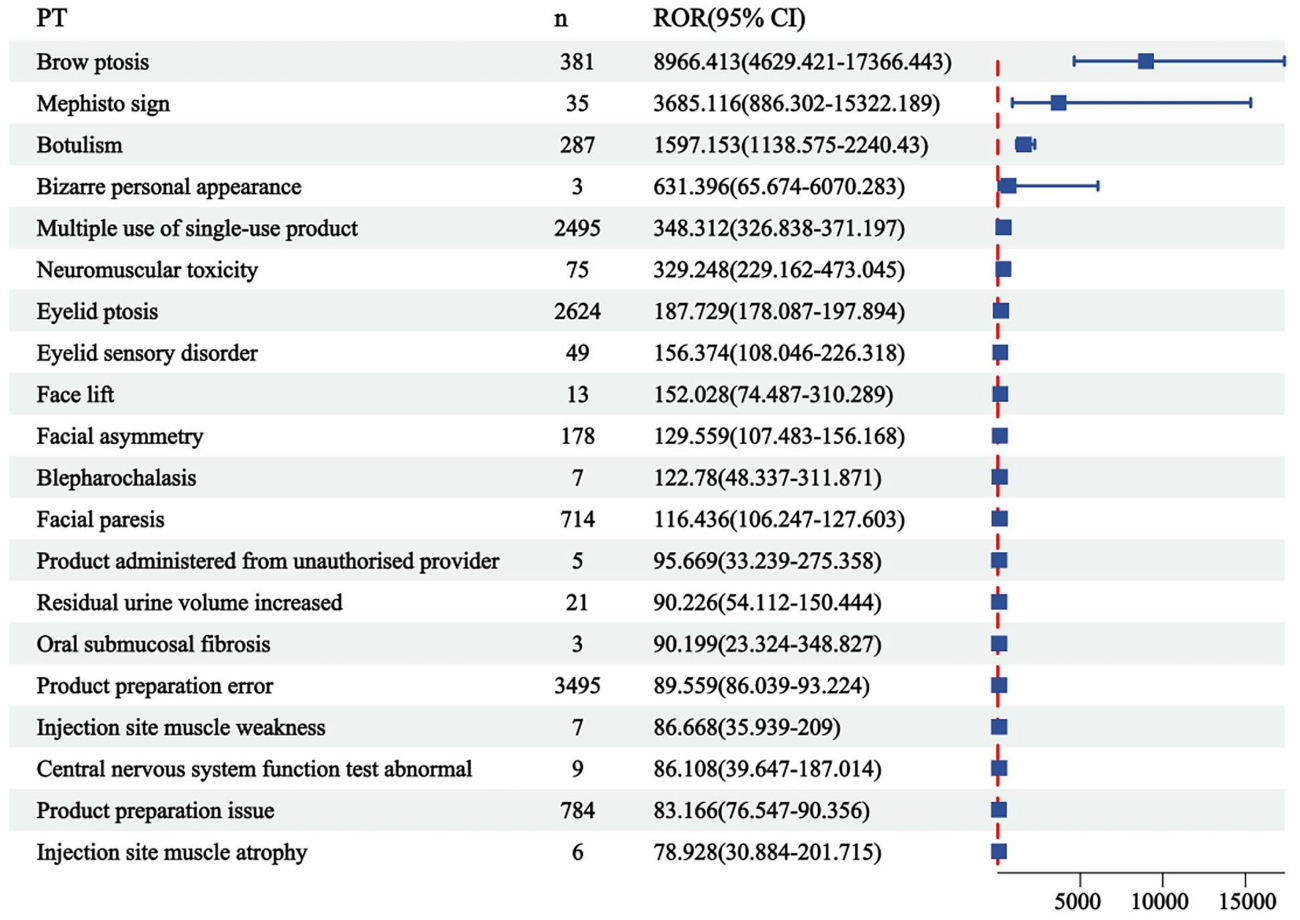
Top 20 signal strengths of reporting odds ratio (ROR) for BoNTA at the preferred terms (PTs) level from FAERS. ROR, reporting odds ratio; CI, confidence interval.

The correlation between PRR and ROR was 1.00, indicating that these two risk indicators were highly correlated. ROR was selected to simplify the analysis process, as shown in Figure 3. Among 325 disproportionately reported events, the top 20 signals with the highest reporting frequency or intensity were analyzed. As illustrated in Figure 4, after excluding non-drug safety issues, such as drug ineffectiveness, off-label use, decreased therapeutic response, and product preparation errors, the five most frequently reported PTs were eyelid ptosis, dysphagia, muscular weakness, blurred vision, and injection site swelling. The ROR values reflect the strength of the association between BoNTA and AEs. The top five PTs based on the signal strength of the ROR were brow ptosis, Mephisto sign, botulism, bizarre personal appearance, and neuromuscular toxicity (Figure 5). The PRR values of the high-frequency and high-intensity signals reported above were significantly higher than the established threshold.

### Analysis of adverse event reports by lowest level term (LLT)

**Figure 6:**
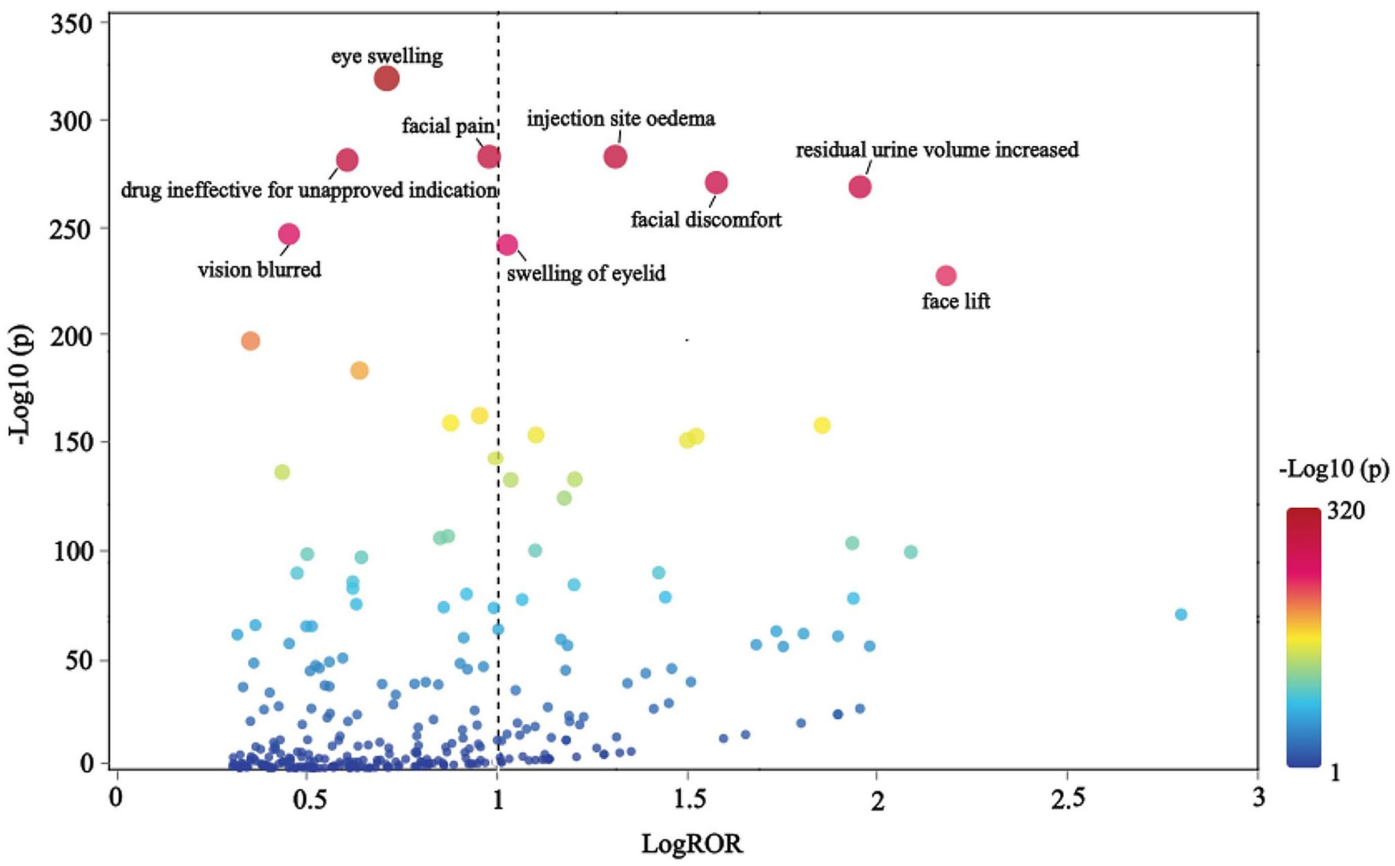
Trend chart of LLT caused by BoNTA from FAERS.

The terms were organized hierarchically across five levels, from broad to specific: SOC, High-Level Group Term (HLGT), High-Level Term (HLT), PT, and Lowest Level Term (LLT), with LLT representing the highest level of specificity. A scatter plot of LogROR versus -Log10(p) is presented in Figure 6, where each point corresponds to an LLT and the size of the dot reflects the number of cases associated with each LLT. An increase in -Log10(p) indicated a statistically significant result. After excluding signals related to non-indication ineffectiveness (e.g., facial wrinkle treatment) and non-drug safety attributes (e.g., injection site swelling), the analysis revealed that BoNTA was associated with additional AEs, including eye swelling, injection site edema, facial pain, facial discomfort, increased residual urine volume, blurred vision, and eyelid swelling.

## Discussion

### Pharmacological action and characteristics of BoNTA-A in the treatment of CP

BoNTA is a complex composed of neurotoxins, hemagglutinin, and non-hemagglutinin proteins, consisting of a heavy chain and a light chain linked by a disulfide bond. The heavy chain contains highly selective binding sites on peripheral cholinergic neurons, enabling the toxin to enter synapses. The light chain is a zinc-dependent peptidase that serves as the primary active component responsible for its toxic effects. It inhibits the calcium-mediated release of acetylcholine at nerve endings, leading to chemical denervation of the muscle. This process disrupts neuromuscular transmission, ultimately resulting in muscle relaxation and reduced tone. The onset of action following local injection typically occurs within 12–72 h, with effects lasting for 3–6 months. Subsequently, new lateral buds and motor end plates form at the nerve endings, restoring their original characteristics and leading to the re-emergence of spasm symptoms. BoNTA does not directly affect axons or peripheral nerves but exhibits a strong specific affinity for synaptosome-associated protein 25 (SNAP-25) related to axonal function. Consequently, the toxin rarely enters the bloodstream or crosses the blood–brain barrier (Delgado et al., 2021; Dorf et al., 2024; Liguori et al., 2023; Tang et al., 2022), ensuring its safety.

### Analysis of the AE report for BoNTA

From the perspective of the reporting timeline for BoNTA AE reports, the overall increase in the number of reports may reflect the growing clinical utilization of BoNTA. As a highly effective drug, BoNTA is widely used in various fields, including aesthetic treatments (e.g., wrinkle removal, masseter hypertrophy, hyperhidrosis), neurological disorders (e.g., spastic CP, blepharospasm, hemifacial spasm), and urinary system diseases (e.g., cystitis, overactive bladder, and benign prostatic hyperplasia) (Salame et al., 2023; Onan et al., 2024). Its usage has been continuously increasing owing to the recognition of its therapeutic efficacy and the growing demand from patients. Hence, the increase in the number of AE reports is likely, to a great extent, a natural consequence of the widespread and growing use of the drug.

The United States, which has the highest number of submissions, highlights its dominance in BoNTA usage and associated AE reporting. This prominence may be attributed to the country’s stringent drug regulatory policies and well-developed AE reporting system. Regarding patient age, the 36–60 age group represented the largest proportion. This may be because individuals in this age range are more likely to pursue cosmetic or medical interventions, increasing their exposure to the drug and, consequently, the likelihood of adverse reactions. Additionally, with advancing age, physiological functions and metabolic capacities of the human body may undergo significant changes. Responses to drugs may also become more sensitive or complex with age. Simultaneously, the number of reports from females significantly exceeded that of males. This disparity could be attributed to the predominant use of BoNTA in cosmetic procedures, which is more commonly sought by the female population. Nevertheless, disparities in age and sex distribution could also be influenced by variations in disease profiles, treatment needs, and patient preferences. These factors highlight the need for further evaluation and validation in clinical practice to comprehensively understand their underlying causes. Finally, serious incidents, including prolonged hospitalization, death, disability, and life-threatening situations, comprised a considerable proportion of reported serious adverse outcomes. This underscores the critical importance of enhancing safety regulations and monitoring the clinical application of BoNTAs to effectively mitigate potential risks.

### Analysis of AE disproportionately reported events for BoNTA

This study analyzed the three levels of AE (SOC, PT, and LLT) in MedDRA and identified that BoNTA tends to induce general disorders and administration site conditions. These include, but are not limited to, local pain, edema, erythema, nodules, and similar reactions. Injection site swelling ranked among the top five disproportionately reported events, primarily associated with BoNTA injection administration and repeated injections. Although these reactions are common, they can significantly impact patient comfort during treatment and pose challenges to treatment compliance. Additionally, eye disorders, another prominent category of AEs, indicate that the use of BoNTA may exert direct or indirect effects on the visual system. Eyelid ptosis, which ranks first in both frequency and intensity, is a notable risk factor caused by the involvement of the upper eyelid. This condition often occurs when BoNTA is injected around the eye or between the eyebrows, allowing the toxin to migrate through the orbital septum and subsequently weakening the levator palpebrae superioris muscle (Borba et al., 2022). Nervous system disorders, the third most prevalent complication of reported AEs, are likely to be linked to the direct impact of BoNTA on the neuromuscular junction. The local effects of BoNTA are expected because the toxin is a local therapy, and a local neuromuscular effect is consistent with the mechanism of action, as both clinical observations and single-fiber electromyography (SFEMG) have consistently demonstrated its strong inhibitory effect on neural impulses. BoNTA injections can diffuse beyond the target area, potentially causing muscular weakness, including eyelid ptosis, dysphagia, facial paresis, and brow ptosis (Kouyoumdjian et al., 2020; Eleopra et al., 2020). Injury, poisoning, and procedural complications highlight technical challenges associated with the treatment process. These complications may include accidental injury to the blood vessels, nerves, or surrounding tissues, as well as toxicity symptoms resulting from drug overdose or improper administration. In rare instances, inaccurate dosing or excessive use of BoNTA may result in symptoms and signs resembling botulism, such as blurred vision and dysphagia. These effects arise from the excessive inhibition of BoNTA at the neuromuscular junction, causing impaired muscle contraction and an inability to properly regulate muscle function (Machamer et al., 2022, 2023). Furthermore, it has been observed that BoNTA can also impact facial appearance, resulting in the Mephisto sign and a peculiar personal appearance. One study reported the occurrence of the Mephisto sign following BoNTA treatment for chronic migraine prevention. This sign is characterized by the outer end of the eyebrow being positioned higher than the inner end, which may create a fearful expression and could be a side effect of BoNTA injection into the frontalis muscle (Cho et al., 2013).

### Clinical implications for risk prevention and control in CP treatment

The risk signals of adverse events identified in this study provide a clear direction for the safe management of CP patients receiving BoNTA treatment. The results showed that dysphagia, muscle weakness, and local swelling were particularly prominent, and their mechanism of occurrence was closely related to the local diffusion and systemic effects of toxins. This suggests that a multi-level risk prevention and control system needs to be established in clinical practice. First, the principle of individualized dose based on body weight should be strictly followed, and the total dose of a single treatment should be reasonably controlled to reduce the risk of systemic reactions. Second, the accuracy of injection technology is crucial, and it is recommended to operate with the assistance of positioning techniques such as ultrasound guidance to ensure that BoNTA accurately acts on the target muscle group and avoids unexpected effects on important functional muscles, such as the swallowing and respiratory muscles, to the greatest extent. In addition, for patients who receive repeated injections, it is necessary to dynamically evaluate their cumulative dose and local tissue response and be wary of the risk changes that may be brought about by the immune response and effect superposition. Finally, the combination of drug injection and systematic rehabilitation training gives full play to the advantages of muscle spasm treatment, and constructs a treatment plan with functional safety as the core through full monitoring and early intervention.

### Limitations of the study

This study has the following limitations. First, as a study based on a spontaneous reporting system, its data are susceptible to incomplete reporting, reporting bias (e.g., overreporting), and missing information (e.g., incomplete age, sex, injection dose, injection site, and frequency of injections). Second, the FAERS data cannot rule out the confusion of concomitant medications and the underlying diseases of patients. The mined signals are only statistically correlated, and the exact causal relationship needs to be further studied and verified. Third, the high concentration of reporting sources in the United States limits the extrapolation of the results, and caution should be exercised when applying it to other regions. Fourth, this study uses ROR and PRR methods for extensive signal scanning. Although the consistency of the main signals in the two methods has been verified, Bayesian methods such as BCPNN have not been introduced, and more complex models can be used for in-depth analysis of specific rare events in the future.

## Conclusion

Based on comprehensive signal mining of BoNTA using the FAERS database, this study systematically revealed a broad spectrum of adverse event risks involving multiple organ systems in real-world applications. Among these, ptosis, dysphagia, and muscle weakness were identified as high-frequency and prominent risks. These findings provide important warnings and insights into the safe use of BoNTA for treating cerebral palsy. The core risks of BoNTA are closely related to its pharmacological mechanisms of action. Therefore, in clinical practice, to ensure that the therapeutic benefits outweigh the potential risks, the high-frequency and high-intensity signals identified in this study should be used as key indicators for safety monitoring. Enhancing the accuracy of injection techniques, strengthening post-treatment follow-up, and improving safety education for patients and caregivers can facilitate early detection and timely management of potential adverse reactions. Such measures can help maximize medication safety in patients with CP while fully leveraging the therapeutic advantages of BoNTA.

## Data Availability

The original contributions presented in the study are included in the article/supplementary material, further inquiries can be directed to the corresponding author.

## Footnotes

^1^ https://openvigil.sourceforge.net/

## References

[ref1] Borba A. Matayoshi S. Rodrigues M. (2022). Avoiding complications on the upper face treatment with botulinum toxin: a practical guide. Aesth. Plast. Surg. 46, 385–394. doi: 10.1007/s00266-021-02483-1, PMID: 34341857 PMC8328485

[ref2] Cho E. S. Hwang J. Y. Kim S. T. (2013). A proposal to prevent the "Mephisto sign" side effect of botulinum toxin type A injection in chronic migraine. Yonsei Med. J. 54, 1542–1544. doi: 10.3349/ymj.2013.54.6.1542, PMID: 24142664 PMC3809864

[ref3] Delgado M. R. Tilton A. Carranza-Del Río J. Dursun N. Bonikowski M. Aydin R. . (2021). Efficacy and safety of abobotulinumtoxinA for upper limb spasticity in children with cerebral palsy: a randomized repeat-treatment study. Dev. Med. Child Neurol. 63, 592–600. doi: 10.1111/dmcn.14733, PMID: 33206382 PMC8048784

[ref4] Dorf S. R. Fonseca A. R. Sztajnbok F. R. Oliveira T. R. D. Basttistella L. R. (2024). The state of the art in therapeutic administration of botulinum toxin in children with cerebral palsy: an integrative review. Rev. Paul. Pediatr. 42:e2023093. 38537033 10.1590/1984-0462/2024/42/2023093 PMC10962635

[ref5] Eleopra R. Rinaldo S. Montecucco C. Rossetto O. Devigili G. (2020). Clinical duration of action of different botulinum toxin types in humans. Toxicon 179, 84–91. doi: 10.1016/j.toxicon.2020.02.020, PMID: 32184153

[ref6] Jonsson U. Eek M. N. Sunnerhagen K. S. Himmelmann K. (2019). Cerebral palsy prevalence, subtypes, and associated impairments: a population-based comparison study of adults and children. Dev. Med. Child Neurol. 61, 1162–1167. doi: 10.1111/dmcn.14229, PMID: 30950519

[ref7] Khandaker G. Muhit M. Karim T. Smithers-Sheedy H. Novak I. Jones C. . (2019). Epidemiology of cerebral palsy in Bangladesh: a population-based surveillance study. Dev. Med. Child Neurol. 61, 601–609. doi: 10.1111/dmcn.14013, PMID: 30394528

[ref8] Klein C. Gouron R. Barbier V. (2024). Effects of botulinum toxin injections in the upper limbs of children with cerebral palsy: a systematic review of the literature. Orthop. Traumatol. Surg. Res. 110:103578. doi: 10.1016/j.otsr.2023.103578, PMID: 36754169

[ref9] Koman L. A. Mooney J. F. 3rd Smith B. Goodman A. Mulvaney T. (1993). Management of cerebral palsy with botulinum-a toxin: preliminary investigation. J. Pediatr. Orthop. 13, 489–495. doi: 10.1097/01241398-199307000-00013, PMID: 8370782

[ref10] Kouyoumdjian J. A. Graça C. R. Oliveira F. N. (2020). Jitter evaluation in distant and adjacent muscles after botulinum neurotoxin type a injection in 78 cases. Toxins 12:549. doi: 10.3390/toxins12090549, PMID: 32867187 PMC7551434

[ref11] Lee S. Robinson K. Lodge M. Theroux M. Miller F. Akins R. Jr. (2021). Resistance to neuromuscular blockade by Rocuronium in surgical patients with spastic cerebral palsy. J Pers Med. 11:765. doi: 10.3390/jpm11080765, PMID: 34442409 PMC8400439

[ref12] Li D. Chai S. Wang H. Dong J. Qin C. Du D. . (2023). Drug-induced QT prolongation and torsade de pointes: a real-world pharmacovigilance study using the FDA adverse event reporting system database. Front. Pharmacol. 14:1259611. doi: 10.3389/fphar.2023.1259611, PMID: 38186652 PMC10771307

[ref13] Liguori S. Young V. M. Arienti C. Pollini E. Patrini M. Gimigliano F. . (2023). Overview of Cochrane systematic reviews for rehabilitation interventions in individuals with cerebral palsy: a mapping synthesis. Dev. Med. Child Neurol. 65, 1280–1291. doi: 10.1111/dmcn.15572, PMID: 36908077

[ref14] Machamer J. B. Vazquez-Cintron E. J. O'Brien S. W. Kelly K. E. Altvater A. C. Pagarigan K. T. . (2022). Antidotal treatment of botulism in rats by continuous infusion with 3,4-diaminopyridine. Mol. Med. 28:61. 35659174 10.1186/s10020-022-00487-4 PMC9164507

[ref15] Machamer J. B. Vazquez-Cintron E. J. Stenslik M. J. Pagarigan K. T. Bradford A. B. Ondeck C. A. . (2023). Neuromuscular recovery from botulism involves multiple forms of compensatory plasticity. Front. Cell. Neurosci. 17:1226194. 37650071 10.3389/fncel.2023.1226194 PMC10463753

[ref16] Onan D. Farham F. Martelletti P. (2024). Clinical conditions targeted by OnabotulinumtoxinA in different ways in medicine. Toxins 16:309. doi: 10.3390/toxins16070309, PMID: 39057949 PMC11280961

[ref17] Paul S. Nahar A. Bhagawati M. Kunwar A. J. (2022). A review on recent advances of cerebral palsy. Oxidative Med. Cell. Longev. 2022:2622310. 10.1155/2022/2622310 PMC9356840 35941906

[ref18] Salame N. Eber A. E. Dover J. (2023). DaxibotulinumtoxinA-lanm (Daxxify™): a comprehensive overview. Skin Therapy Lett. 28, 1–3, PMID: 37440610

[ref19] Selinger A. J. Cavallin N. A. Yanai A. Birol I. Hof F. (2022). Template-directed synthesis of bivalent, broad-spectrum hosts for neuromuscular blocking agents. Angew. Chem. Int. Ed. Engl. 61:e202113235. 34889016 10.1002/anie.202113235

[ref20] Shi J. Liu X. Jiang Y. Gao M. Yu J. Zhang Y. . (2024). CAR-T therapy pulmonary adverse event profile: a pharmacovigilance study based on FAERS database (2017-2023). Front. Pharmacol. 15:1434231. 39234101 10.3389/fphar.2024.1434231 PMC11371680

[ref21] Tang H. Peng T. Yang X. Liu L. Xu Y. Zhao Y. . (2022). Plasma Metabolomic changes in children with cerebral palsy exposed to botulinum neurotoxin. J. Proteome Res. 21, 671–682. doi: 10.1021/acs.jproteome.1c00711, PMID: 35018779

[ref22] te Velde A. Morgan C. Novak I. Tantsis E. Badawi N. (2019). Early diagnosis and classification of cerebral palsy: an historical perspective and barriers to an early diagnosis. J. Clin. Med. 8:1599. doi: 10.3390/jcm8101599, PMID: 31623303 PMC6832653

[ref23] Vitrikas K. Dalton H. Breish D. (2020). Cerebral palsy: an overview. Am. Fam. Physician 101, 213–220. 32053326

[ref24] Walhain F. Desloovere K. Declerck M. Van Campenhout A. Bar-On L. (2021). Interventions and lower-limb macroscopic muscle morphology in children with spastic cerebral palsy: a scoping review. Dev. Med. Child Neurol. 63, 274–286. doi: 10.1111/dmcn.14652, PMID: 32876960

